# Impact of cold plasma processing on major peanut allergens

**DOI:** 10.1038/s41598-020-72636-w

**Published:** 2020-10-12

**Authors:** Harshitha Venkataratnam, Orla Cahill, Chaitanya Sarangapani, P. J. Cullen, Catherine Barry-Ryan

**Affiliations:** 1 School of Food Science and Environmental Health, College of Sciences and Health, Technological University Dublin, Cathal Brugha Street, Dublin 1, Republic of Ireland; 2 Centre for Advanced Food Enginomics, School of Chemical and Biomolecular Engineering, The University of Sydney, Sydney, Australia; 3 Plasmaleap Technologies, Merewether Building, City Road, Sydney, Australia

**Keywords:** Biochemistry, Biotechnology

## Abstract

Cold plasma is emerging as a novel food processing technology, with demonstrated efficacies for microbial inactivation and residual chemical dissipation of food products. Given the technology’s multimodal action it has the potential to reduce allergens in foods, however data on the efficacy and mechanisms of action are sparse. This study investigates the efficacy of cold plasma on major peanut allergens (Ara h 1 and Ara h 2). For this purpose, dry, whole peanut (WP) and defatted peanut flour (DPF) were subjected to an atmospheric air discharge using a pin to plate cold plasma reactor for different treatment durations. With increases in plasma exposure, SDS-PAGE analysis revealed reduced protein solubility of the major peanut allergens. Alterations in allergenicity and structure of Ara h 1 and Ara h 2 were examined using ELISA and circular dichroism (CD) spectroscopy. Competitive ELISA with proteins purified from plasma treated WP or DPF revealed reduced antigenicity for both Ara h 1 and Ara h 2. The highest reduction in antigenicity was 65% for Ara h 1 and 66% Ara h 2 when purified from DPF. Results from CD spectroscopy analysis of purified proteins strongly suggests the reduction in antigenicity is due to modifications in the secondary structure of the allergens induced by plasma reactive species. Cold plasma is effective at reducing peanut protein solubility and causes changes in allergen structure leading to reduced antigenicity.

## Introduction

Food allergy is a global health concern. The World Allergy Organisation’s epidemiological survey estimated that there are between 220–250 million people suffering from a food allergy condition. Approximately 8% of children and 3–4% of adults are affected by these food allergies^1,2^. More than 150 million people living in Europe are affected by food allergies, and 44% of adults who live in Britain have one form of allergy^3^. Over 170 foodstuffs are believed to cause allergy^4^. Due to their worldwide cultivation and consumption, legumes are one of the leading causes of allergic reactions from food. Legumes are also considered as one of the preferred vegetarian proteins throughout the world^2^. Peanuts and soybean belong to the *Leguminosae* family and are two of the eight ingredients that cause important food allergies in the US and Europe^5^. Peanut allergy is often the most severe and it is the leading cause of deadly anaphylaxis. Peanut proteins are the causative agent for IgE-mediated food allergies. The World Health Organisation (WHO) and the International union of immunological Societies’ Allergen Nomenclature subcommittee currently recognise 17 peanut allergens^6^. Out of these 17 allergens Ara h 1, Ara h 2, Ara h 3, Ara h 6 and Ara h 7 are the five major allergens as they account for a majority of the effector activity found in crude peanut extracts^7^. These allergens are grouped into protein families and super families. Ara h 2, Ara h 6 and Ara h 7 are 2S albumins and belong to the prolamin superfamily with a molecular weight between 14- 20 kDa. Ara h 1 and Ara h 3 fall within the cupin superfamily and are further classified in the vicilin (Ara h 1) and legumin (Ara h 3) families with molecular weights ranging between 60 to 65 kDa^8,9^.

Currently several novel food processing technologies are being explored to improve the quality, shelf-life and sensory attributes of foods. During processing, food proteins may undergo modifications which can minimize the risk associated with food allergies. Given the risks associated with these products there is a need to develop processing technologies which can eliminate/modify the allergen proteins for the preparation of hypoallergenic or nonallergenic food.

To overcome the allergenic potential of peanut, several strategies have been reported which include; enzymatic proteolysis, genetic modification and physical methods^4^. Most of the hypoallergenic food currently available in the market are manufactured primarily by enzymatic proteolysis. Although this approach has the ability to alter the sensitivity, it can have negative impacts on the organoleptic characteristics of food products^10^. The use of genetically modified food remains controversial in many countries^11^. Thermal processing either by humid or dry treatments can be effective but may also impact the sensory and nutritional value of the food. Therefore, non-thermal processing technologies such as atmospheric cold plasma, which can induce changes in proteins and potentially mitigate allergenicity whilst retaining the organoleptic properties of food are currently being investigated.

Recently cold plasma has emerged as a novel food processing technology offering increased safety profiles and extended shelf life for food products^12^. Electric discharges in atmospheric air generate large quantities of reactive species^13^. In air over 100 different species may be generated; including key species such as ozone, hydroxyl radicals and hydrogen peroxide. These species can react with food biomolecules leading to changes in their structural and functional properties. Cold plasma technology can be considered as a green and sustainable technology as it does not leave any residues and uses low input energies^14,15^. Applications of cold plasma for microbial decontamination and modification of food packaging surfaces have already been established^16^. Other applications of cold plasma in the agri-food industry include; modification of food surfaces, germination of food grains^17^, mycotoxin and pesticide degradation and wastewater treatment^18^. Only a small number of studies to date have reported the effects of cold plasma on food allergens^19,20^ and although the cold plasma mechanism of action on allergen has not been elucidated in detail, it is has been assumed that the reactive species generated alters the epitope structure^21^. Several authors have reported possible changes in the protein structure of the PPO and POD enzymes due to plasma treatment^22^. Recently, Meinlschmidt et al.^20^ and Venkataratnam et al.^23^ reported changes in the immunoreactivity of soy and allergenicity of peanut after cold plasma treatment. However, data on the effect of cold plasma on the allergenicity of major peanut allergens are sparse. The objective of this study is to investigate the potential of cold plasma in reducing the allergenicity of major peanut allergens Ara h 1 and Ara h 2 using a novel large volume pin-to-plate atmospheric plasma reactor.

## Material and methods

### Materials

Raw peanuts (*Arachis hypogaea*) were purchased from a local market in Dublin, Ireland and stored at room temperature. All reagents and chemicals used in this study were of analytical grade and obtained from Sigma-Aldrich, Dublin, Ireland. For preparation of defatted peanut flour, peanuts were ground using a pestle and mortar and defatted by addition of hexane for 5 h at 4 °C.

### Cold plasma treatment

**Figure 1:**
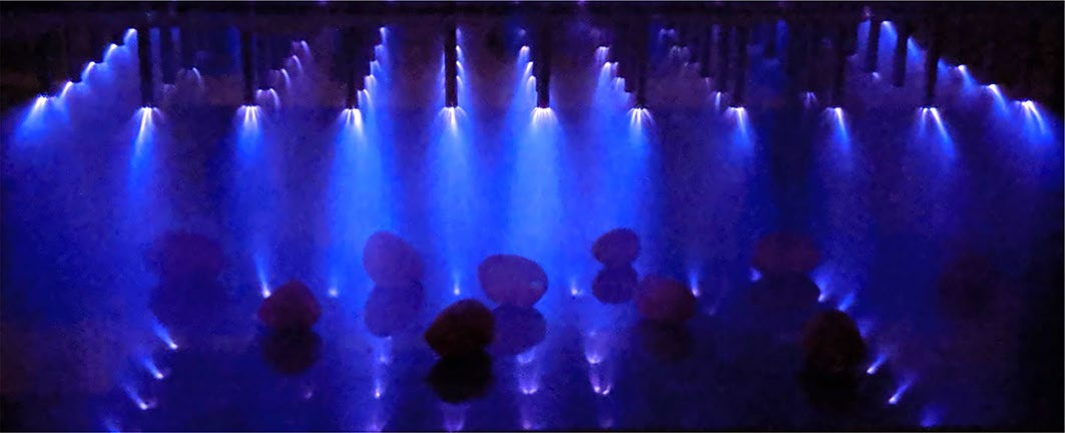
Large volume plasma discharge in atmospheric air using an 88-pin electrode.

For this study, a novel large gap pin-to-plate plasma reactor was employed (Leap100, Plasma Leap Technologies, Sydney, Australia). A detailed description of the working principle of this power source and pin reactor has been provided previously^24^. The reactor uses two steel plates as electrodes with a high voltage electrode composed of a pin array (11 × 8) and a flat plate ground electrode. The electrode pins placed to create a slight convex pattern that promotes a homogeneous plasma discharge across the array with the core pins nearer to the ground electrode. In this study, the mean distance between the bottom of the pins was maintained at 7 cm (Fig. 1). The high-voltage power supply (Leap100, PlasmaLeap Technologies, Sydney, Australia) used to generate the plasma discharge in atmospheric air was set to a resonant frequency of 52 kHz and a discharge voltage of 32 kV. The duty cycle was maintained at 118 s with a discharge frequency of 1 kHz. For each experiment, 10 g of each deshelled whole peanut (WP) with skin and ground peanut flour i.e., defatted peanut flour (DPF) were added to a petri dish separately and placed between two steel plates for plasma treatment (Fig. 1).

### Extraction and purification

After plasma treatments, Ara h 1 and Ara h 2 from the peanut samples (WP and DPF) were extracted and purified using the procedures of Maleki et al*.*^25^ and Koppelman et al.^26^ with slight modifications. Briefly, crude protein extract from WP and DPF was extracted using extraction buffer (50 mM Tris, 200 mM NaCl, and 1 mM EDTA at pH 8.3) for 2 h at 4 °C. The samples were centrifuged at 12,000 g for 15 min, the supernatants were subjected to ammonium sulphate precipitation (0–40%, 40–70% and 70–100%) and the protein pellet was collected after centrifugation. The collected protein has been further resolubilized in buffer at pH 8.3 without NaCl, desalted by dialysis/ultrafiltration against buffer overnight in the ratio 1:40. This was loaded on to a High Prep Q column and eluted with a linear salt gradient (40–300 mM NaCl). Fractions were collected and Sodium Dodecyl Sulphate polyacrylamide gel electrophoresis (SDS-PAGE) was run at reducing conditions. Protein concentration was checked at every step using the Bradford assay. The purified Ara h 1 and Ara h 2 was then used to study the secondary structure of the protein.

### Electrophoresis

The SDS-PAGE was performed using 12% hand-cast gels the procedures were slightly modified according to earlier studies^27^. Proteins were reduced and denatured using sample buffer. The samples were heated at 100 °C, for 5–7 min, prior to loading. Pre-stained molecular marker obtained from Fisher Scientific (Dublin, Ireland) was used as a reference. Electrophoresis was performed at a constant voltage of 120 V using a tris glycine buffer. Rapid staining was performed according to Studier et al.^28^. Gels were suspended in 50 mL of 50% ethanol, 10% acetic acid and 40% water, heated to almost boiling in a microwave oven and rocked on a shaker until the gel shrinks. The liquid was discarded and suspended in 50 mL of 5% ethanol, 7.5% acetic acid and 0.25% coomassie blue in ethanol. The gel was again heated to boiling in a microwave and placed on a rocker. The gels were visualized within 45 min.

### Immunoblotting

SDS-PAGE gels were prepared, as described in Sect. 2.4 and the proteins separated were transferred to nitrocellulose membrane at a constant voltage of 20 V for 1 h. Immunoblotting was performed according to Venkataratnam et al.^23^ with slight modifications. The nitrocellulose membrane were then blocked with 5% skim milk in phosphate-buffer saline containing 2% Tween 20 (PBST) for 2 h at room temperature, followed by overnight incubation with rabbit anti-Ara h 1 (1:5,000 in PBST, Indoor Biotechnologies) and rabbit anti-Ara h 2 (1:5,000 in PBST, Indoor Biotechnologies) at 4 °C. Subsequently, the membrane was washed thrice using phosphate-buffer saline (PBS), followed by incubation with goat anti-rabbit conjugated with horseradish peroxidase (HRP) (1:8,000, Sigma Aldrich, Dublin, Ireland) for 30 min at room temperature. After further washing, the membranes were incubated with an enhanced chemiluminescent luminol substrate for 1 min and the images were observed on C-diGit blot scanner (LI-COR).

### Competitive enzyme linked immune sorbent assay (ELISA)

Competitive ELISA was performed according to Schmitt et al.^29^ The IgG-binding abilities of cold plasma-treated WP and DPF for both Ara h 1 and Ara h 2 were tested using competitive ELISA. 100 μL of purified native Ara h 1 and Ara h 2 (0.1 μg/mL) were added to every well of a 96-well ELISA plate, and coated in coating buffer (0.1 M NaHCO_3_, pH 9.6) followed by incubation overnight at 4 °C. After incubation, the wells were washed with phosphate-buffer saline tween 20 (PBST) thrice. In order to block non-specific binding, 200 μL of 3% bovine serum albumin in PBST was added to each well and incubated for 2 h at 37 °C. The peanut extracts were mixed with equal volumes of rabbit Anti-Ara h 1 (1:5,000) and rabbit Anti-Ara h 2 (1:5,000) and incubated for 30 min at 37 °C. This mixture was added into the coated plates and further incubated for 1 h at 37 °C. After further washing, 100 μL of goat anti-rabbit conjugated with HRP (1:7,000) were then added to each well, followed by incubation for 30–45 min at 37 °C for the detection of bound immunogen. The plates were further washed, and the colour was developed by adding 100 μL of 3,3′,5,5′-tetramethylbenzidine to each well and incubated for 15 min at room temperature. The reaction was terminated using 100 μL of 1 N hydrochloric acid and the absorbance of processed and unprocessed samples were read at 450 nm with a plate reader.

### Secondary structure determination

CD spectroscopy was performed according to the method of Venkataratnam et al.^23^ with slight modifications. Conformational changes in the secondary structure were determined using Far UV (190–240 nm) circular dichroism (CD) spectra. The far UV-spectra were obtained with a JASON Model J-810 spectropolarimeter on 0.1 mg/mL protein solutions using a 1 mm path length quartz cell, at a rate of 100 nm/min and band width of 1.0 nm. Purified protein obtained from cold plasma treated (WP and DPF) and control Ara h 1 and Ara h 2 were desalted using centrifugal filters into Milli-Q water and immediately used in CD measurements. A CD spectrum of Milli-Q water was obtained for background purpose and subtracted from each spectra. The secondary structure composition was calculated by Dichroweb server (program: CDSSTR; reference set: SET 7 optimized for 190–240 nm).

### Statistical analysis

Statistical analysis was performed using SPSS software (IBM statistical analysis Version 19), with the data analysed by one-way ANOVA. The significance among the samples was compared at *P* < 0.05 by the least significant difference post-hoc comparison. All the tests were performed in duplicate and the average of the tests are represented.

## Results and discussion

### SDS-PAGE

**Figure 2:**
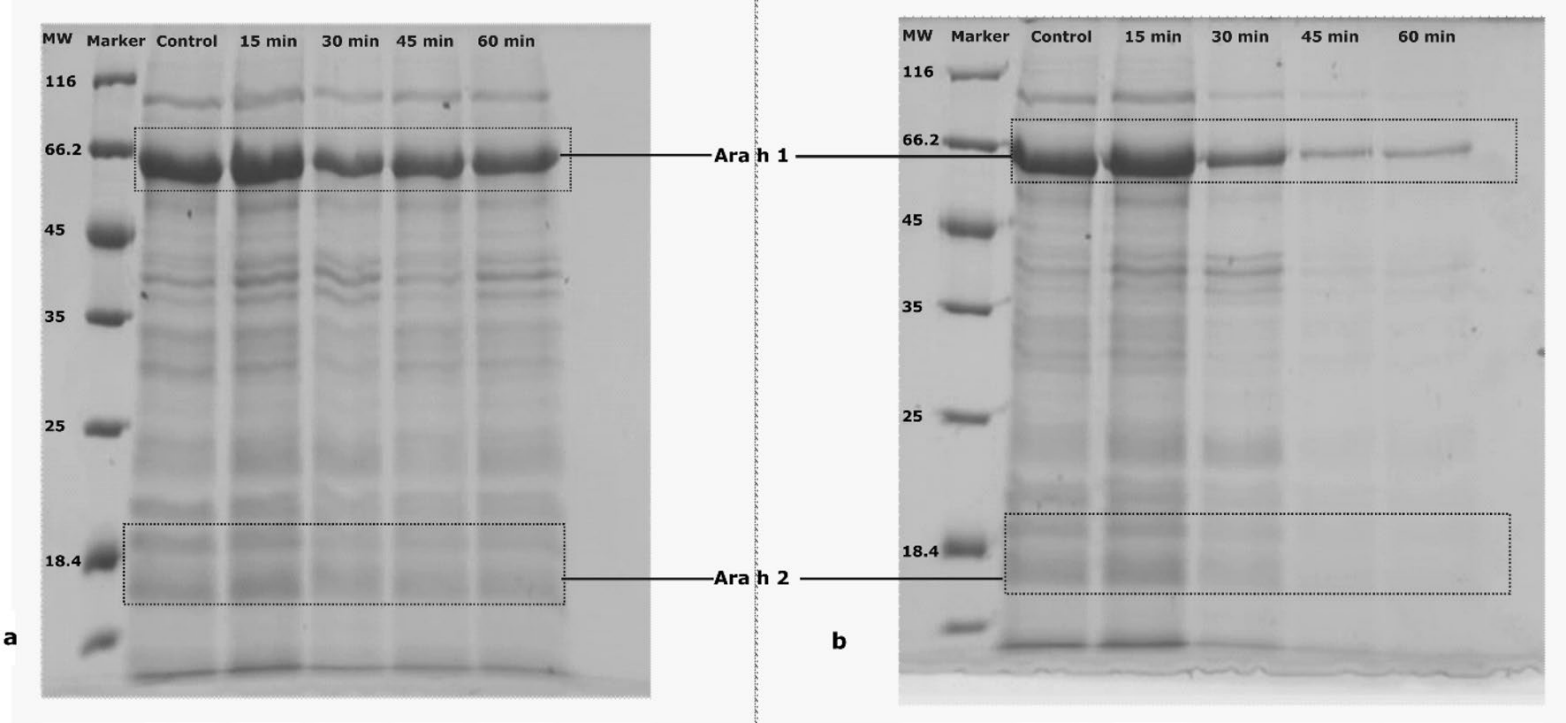
SDS PAGE analysis of Whole peanut (**a**) and defatted peanut flour (**b**) before and after plasma treatment.

The effects of the cold plasma treatment on peanut allergen was assessed by SDS-PAGE to visualize the changes in the allergen content. For this purpose, protein concentrations of 2 mg/mL were combined with sample buffer with equal amounts of total protein (10 µL) loaded for each sample. Figure 2 shows the relative band intensities of the allergen proteins before and after plasma treatment. It is observed from Fig. 2 that all the bands ranged from molecular weight (MW) 11 to 65 kDa. The strong bands identified at 63 kDa corresponds to Ara h 1 and the triplet 18, 19 and 20 kDa corresponds to Ara h 2. As observed from Fig. 2 (a) there were no changes in the intensity with increases in treatment time of up to 15 min for either Ara h 1 or Ara h 2 for WP, however, with further increase in treatment time slight changes in the intensities were observed when compared to the control. The SDS-PAGE analysis of DPF showed a profound change in band intensities when compared to WP. As it can be observed form Fig. 2b after 60 min of treatment time the intensity of Ara h 1 reduced and the band corresponding to Ara h 2 doublet was faint. The difference in the observed effects of plasma on WP and DPF is may be due to changes in food matrix, surface characteristics and the nature of interaction with other food components, including carbohydrates and lipids. Van Wijk et al.^30^ investigated the influence of the food matrix on the immune responses to food proteins. These authors hypothesized that the food body, consisting of fats, carbohydrates, and other proteins, may affect the allergenic potential of proteins. The variation in antigenicity of WP and DPF is that the WP have medium protein concentrations but high levels of fat. These fats may protect proteins during the digestion process/treatment process, or they may affect the allergenicity of a protein. Moreover, the polysaccharide–protein and protein–protein and protein-lipid interactions may influence allergen reduction^31^.

The decrease in band intensity following plasma treatment is due to plasma induced crosslinking of proteins^20^. Atmospheric plasma is a good source of reactive species such as atomic oxygen (O), ozone (O_3_), hydroxyl radical (•OH), N_2_, NO, NO_2_, nitric oxide radical (NO•) as well as UV-A and UV-B radiation^32,33^. These species induce changes in protein structure. Several authors have suggested that the faint band in SDS-PAGE might indicate cross-linking proteins, however, there was no cross-linking observed in the gel^20,34^. Tammineedi et al. observed no change in protein of α-casein after subjecting to plasma treatment. Moreover, these authors observed prolonged exposure of cold plasma resulted in reducing band intensity due to increased protein to protein interactions derived during protein unfolding and aggregation. The formation of aggregates and the decreased protein solubility could lead to the disappearance of protein bands as only soluble proteins can pass through the gel smoothly^35^ in the present study only the soluble proteins have been subjected to SDS-PAGE in this study In addition, the hydroxyl and hydrogen radicals (OH• and H^+^), along with the ozone molecule and UV radiation are known to be powerful protein-modifying agents^36^. In a study on Gly m 5, a major soy allergen which is structurally similar to Ara h 1, a decrease in band intensity and changes in protein profile after plasma treatment was observed^20^. Surowsky et al.^22^ also reported changes in the secondary structure of protein for model enzyme solutions. The decrease in the band intensities of WP and DPF could be attributed to plasma induced changes in the protein structure or oxidation of protein resulting in the formation of disulphide bonds. An earlier study using an atmospheric DBD plasma system, resulted in no changes in Ara h 1 for either WP and DPF^23^. Many authors have reported that the effects induced by plasma depends on the system configuration^21,37^. In the present study, a large discharge was induced between the electrodes resulting in a stable arc discharge in air. The device employed facilitated changes in the frequency (100–3,000 Hz), which was set to 1 kHz to maximise the intensity and effect of the discharge.

### Immunoblotting

**Figure 3:**
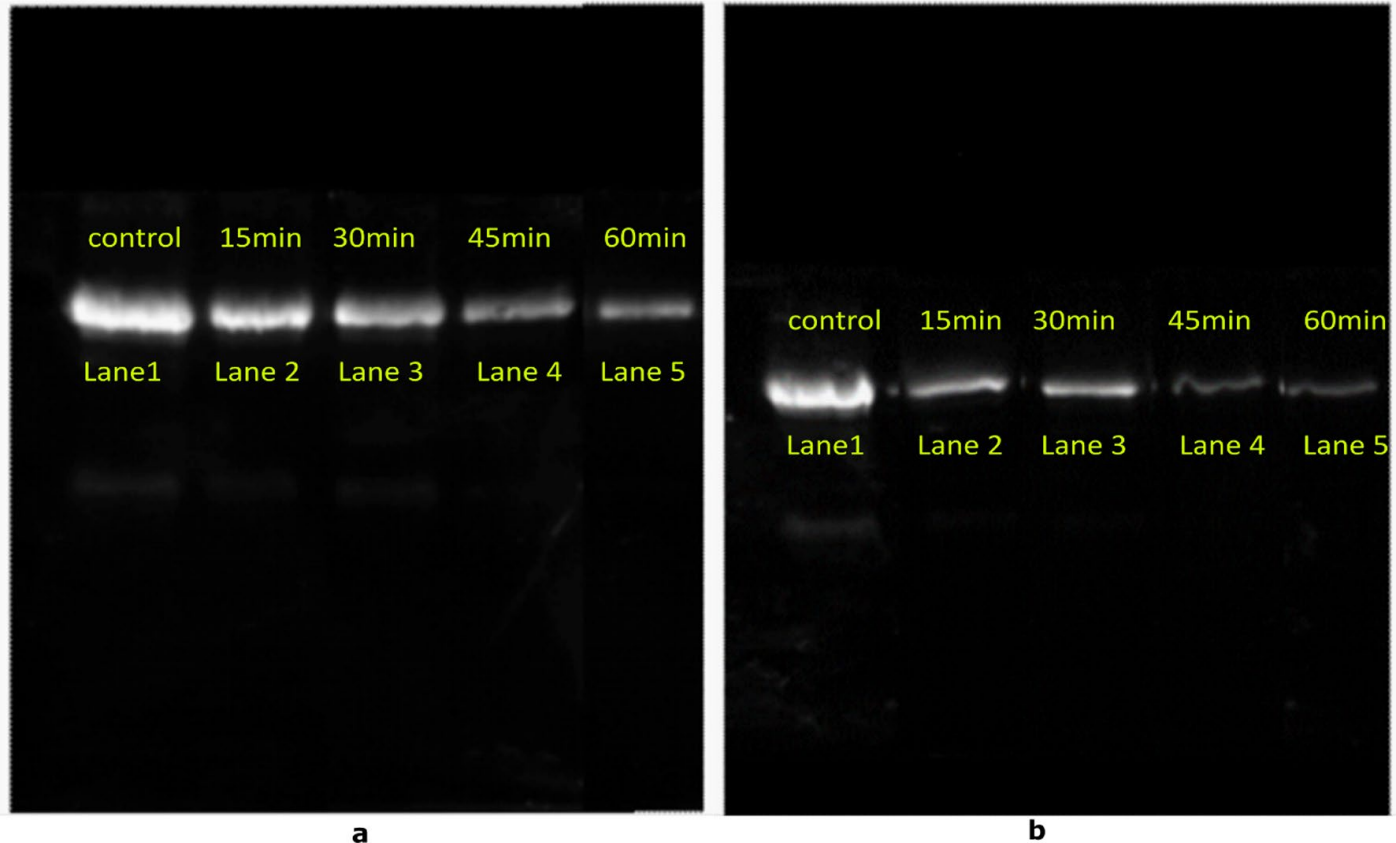
Western blot analysis of Whole peanut (a) and Defatted peanut flour (b) of Ara h 1 (Note: Lane 1: Control; Lane 2 15 min; Lane 3: 30 min; Lane 4: 45 min Lane 5: 60 min).

**Figure 4:**
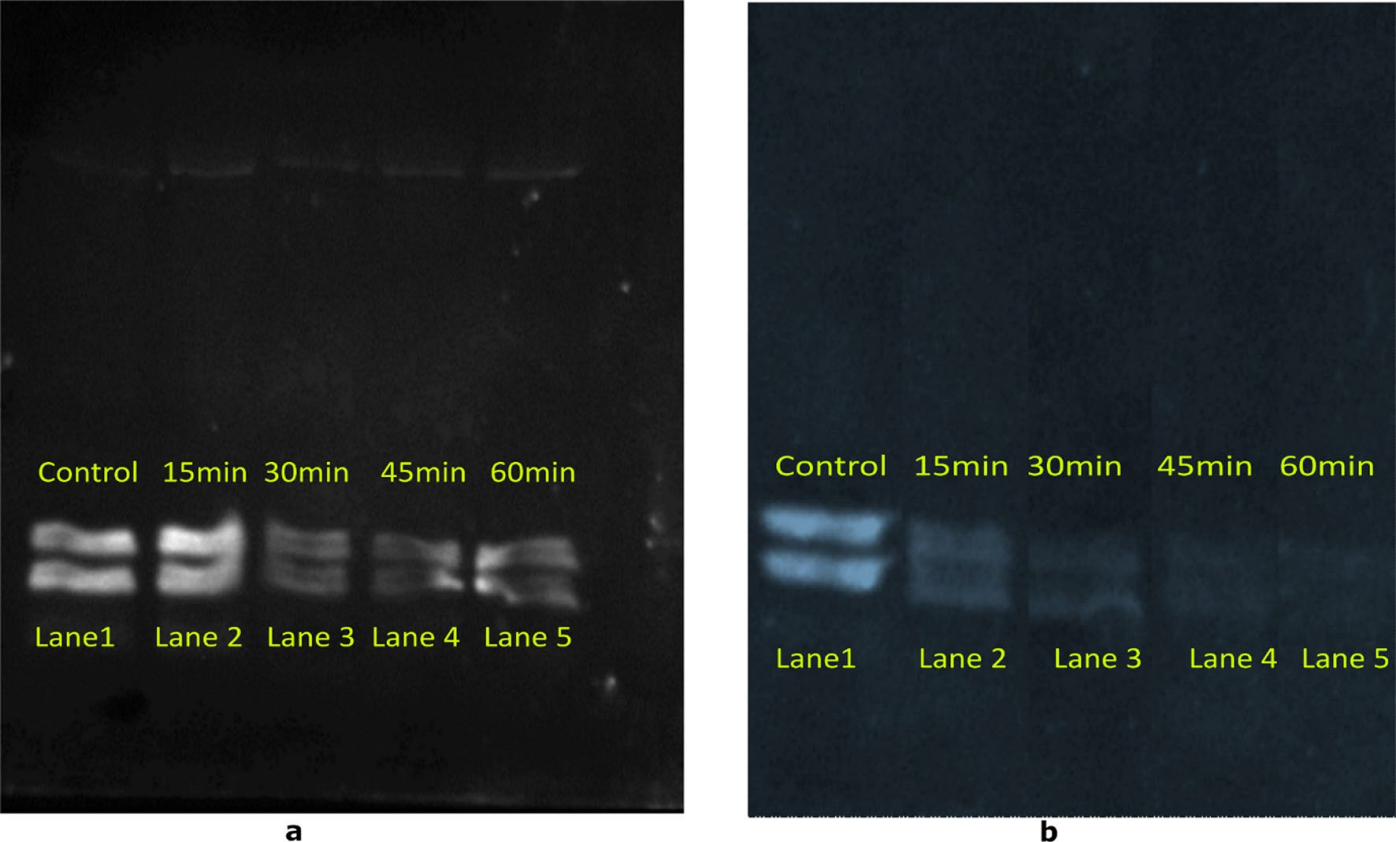
Western blot analysis of Whole peanut (a) and Defatted peanut flour (b) of Ara h 2 (Note: Lane 1: Control; Lane 2 15 min; Lane 3: 30 min; Lane 4: 45 min Lane 5: 60 min).

The changes in the immunoblots for both WP and DPF after cold plasma treatment (15, 30, 45 and 60 min) is presented in Figs. 3 and 4, from which it is evident that the band intensity of Ara h 1 and Ara h 2 decreased with increases in treatment for both WP and DPF compared to the control. With an increase in plasma exposure to 30 min the intensity of the band reduced which could indicate a decrease of solubility of Ara h 1 and Ara h 2. However, this does not mean that there is a decrease in the allergenicity in WP. The reduced band intensity observed in Figs. 3a and 4a for the 45 min and 60 min treated samples suggest a decrease in binding activity due to less protein in the solution. Additionally, it could also be due to the aggregation of proteins. The decrease in the IgG binding activity in DPF is more when compared to the WP, for both Ara h 1 and Ara h 2 and this could be due to the protein-lipid complex in WP. This protein-lipid complex might prevent the reactive species acting on the epitopes. Although, increases in the treatment dose might alter the functionality of the protein-lipid complex^38,39^. In a recent study, low pressure plasma treatment of cashew nut showed a minimal effect in nut composition with increases in oil extractability^40^. These authors indicate the barrier effect of the protein molecules in the upper layer of cashew limited the transition of plasma-generated radical species within the sample leading to no observed changes in the allergenicity after treatment.

In the present study, a decrease in the IgG binding activity for DPF was observed for both Ara h 1 and Ara h 2 as seen in Figs. 3b and 4b, suggesting a decrease in binding. Similar reductions in band intensities were observed by Nooji et al.^39^ with cold plasma treatment of wheat protein extracts. Additionally, Jiang et al.^41^ also reported that plasma efficacy depends on the sample surface area, sample constituents and treatment time. As the treatment time and surface area exposed to the discharge increase, there is more potential for the active species to induce physical or chemical changes. These active species could also mask or change the IgG binding epitope and result in a decrease in the IgG binding pattern^21^. Moreover, sample composition also plays an important role in plasma interactions.

Zhenxing et al.^34^ reported that shrimp allergen was protected from free radical damage due to the presence of lipids in the shrimp muscle during low doses of gamma radiation. Shriver et al.^19^ also reported a lower IgE binding after low plasma doses. The presence of UV light during air plasma treatment may also play a role in lowering the IgE binding. Chromophores e.g., amino acids chains and prosthetic groups present in the proteins are prone to photoreactions^3^. Exposure to UV light makes the protein chromophores absorb the light and cause side-chain oxidation, protein cross-linking and aggregation. This formation of insoluble proteins and backbone fragmentation by UV and other species may cause alteration in IgE binding to the allergen^42^.

### Competitive ELISA

**Figure 5:**
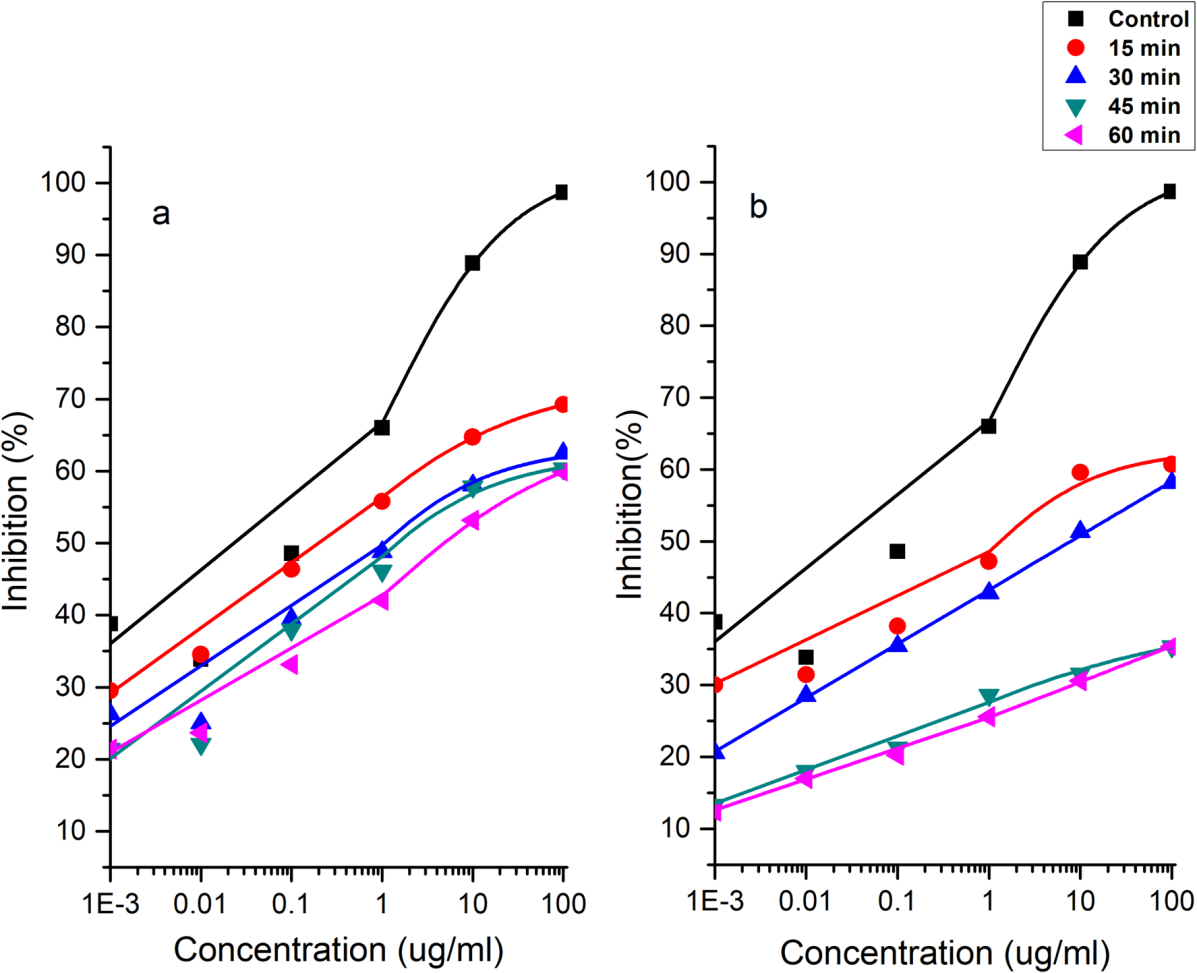
Competitive ELISA of Ara h 1 in cold plasma treated WP (**a**) and DPF (**b**).

**Figure 6:**
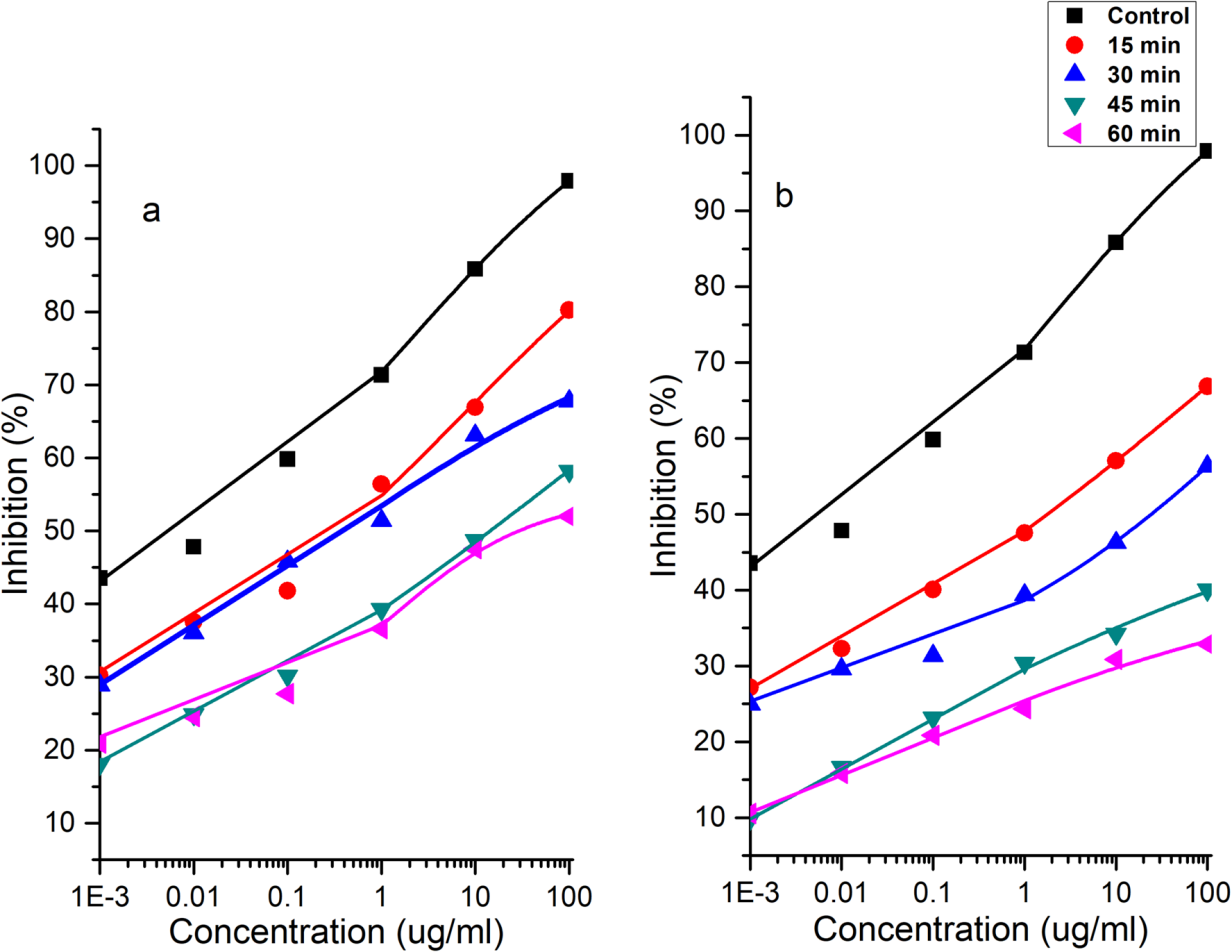
Competitive ELISA of Ara h 2 in cold plasma treated WP (**a**) and DPF (**b**).

Competitive ELISA was used to determine the antigenicity of purified peanut allergens. Ara h 1 and Ara h 2 were extracted and purified from both control (untreated) and cold plasma treated DPF and WP and then used competitive ELISA analysis. Both major allergens, purified Ara h 1 and Ara h 2, were evaluated before and after cold plasma treatment for DPF and WP. Figure 5 shows the antigenicity of Ara h 1 decreased with increase in plasma doses for both WP and DPF Fig. 5a,b. It can be observed from Fig. 6a that Ara h 2 in WP showed a decrease in antigenicity of 18%, 30%, 40% and 46% for 15, 30, 45 and 60 min, respectively. A profound decrease in antigenicity of Ara h 2 was observed for DPF see Fig. 6b when compared to WP. Ara h 2 showed a decrease in antigenicity of 31% and 42% for 15 min and 30 min respectively in DPF. Further increases in treatment doses of DPF to 45 and 60 min showed high reductions of 59% and 66% respectively. Similarly, a decrease in the antigenicity of Ara h 1 was observed for DPF when compared to WP. A decrease of 38%, 41%, 64% and 65% was observed for Ara h 1 in DPF and a decrease of 29%, 36%, 38% and 39.32% was observed for Ara h 1 in WP.

The prominent decrease in antigenicity of Ara h 1 and Ara h 2 in DPF is likely due to the increase in the surface area of DPF compared to WP. Additionally, the difference in the reduction in antigenicity of these allergens are due to changes in the protein structure. Similar reductions were achieved by Venkataratnam et al.^23^ for cold plasma treatment of peanut allergens. Wu et al.^43^ reported that the mechanism of plasma allergen reduction depends on the form of protein structure. The results observed in this study are in agreement with findings from Meinlschmidt et al.^20^ who applied cold plasma for the treatment of soy allergen Gly m 5. These authors achieved a reduction in binding activity (91–100%) with direct and remote cold plasma treatment. Plasma species such as reactive oxygen species (ROS) and reactive nitrogen species (RNS) alter amino acids, resulting in changes in the binding epitopes^44^. During plasma discharges and interactions numerous chemical reactions may occur leading to multiple mechanisms of action and pathways. The primary mechanism of allergen reduction likely involves an alteration of the conformational epitopes by the formation of insoluble aggregates. The SDS-PAGE data supports this mechanism, where only soluble proteins can pass through the gel. Alternatively, the decrease in solubility and the fragmentation could also be the case for alteration of linear epitopes^3^. Besides these Surowsky et al.^22^ also reported that reactive species may cleave amino acids thereby incapacitating the binding sites of antibodies. The dissociative addition of the single hydroxyl to form RSH and RSO• and formation of disulphide bonds can lead to significant effects on protein integrity. These findings show that higher exposures to plasma has a profound effect on the IgE binding proteins which is in agreement with previous studies. Nooji^39^ revealed a 25% decrease in IgE binding after 3 min of plasma treatment. Similarly, Shrivar^19^ reported a reduction in IgE binding to tropomyosin by up to 76% after cold plasma treatment. In addition, a recent study by Ekezie et al.^45^ reported that cold plasma has been efficient in decreasing the allergenicity and antigenicity capacities by 17.6% and 26.8% respectively. Conversely, Tammineedi et al.^46^ observed no significant change in the allergenicity of α-casein and whey solution by indirect atmospheric plasma treatment. The present research found that there may be modifications or biodegradation of protein profiles. However, this tendency seemed to differ according to the nature of the allergen, plasma conditions, its exposure mode and its degree of structural alteration. Most importantly, the plasma parameters employed play an important role in the cleavage of peanut allergens.

### CD spectroscopy

**Figure 7:**
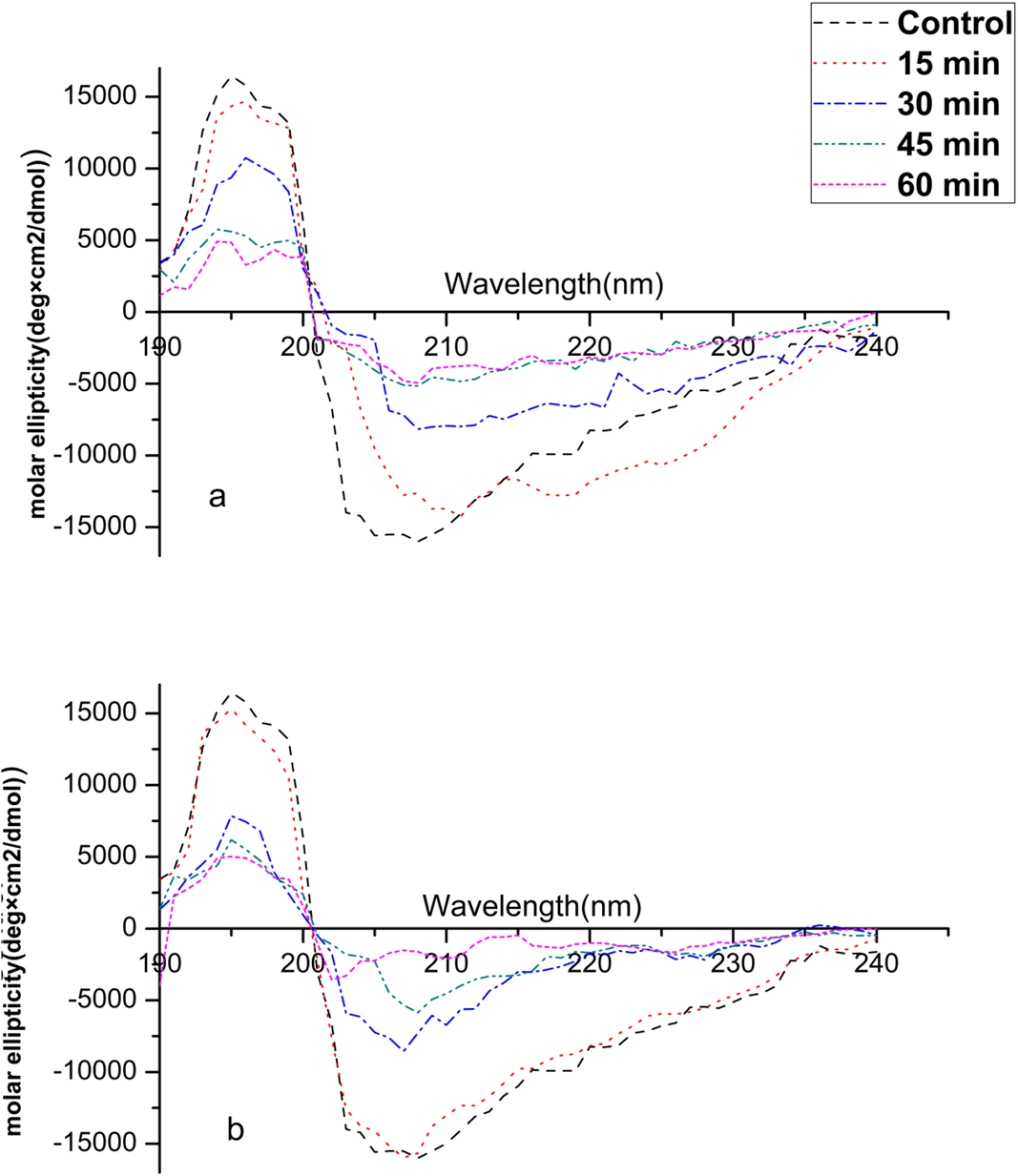
CD spectroscopy of Ara h 1 in WP (**a**) and DPF (**b**).

**Figure 8:**
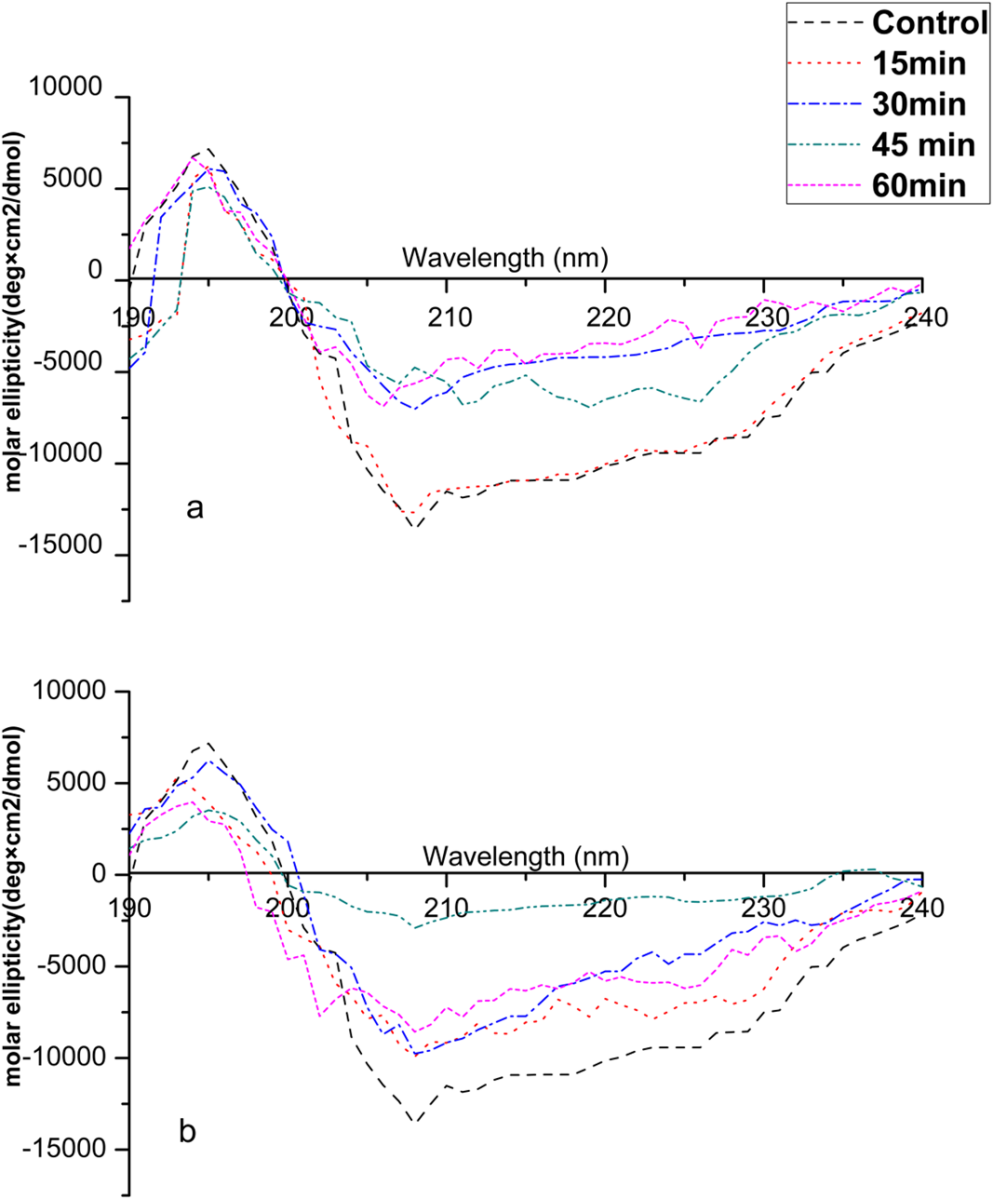
CD spectroscopy of Ara h 2 in WP (**a**) and DPF (**b**).

CD spectral analysis was used to detect modifications in the protein secondary structure. Ara h 1 and Ara h 2 were extracted and purified from both control (untreated) and cold plasma treated DPF and WP and then used for CD spectral analysis. From Fig. 7a,b the spectra of native Ara h 1 showed a characteristic positive peak at 195 nm, indicating the presence of β-sheets and two negative peaks (∼ 208 nm and 222 nm) confirming the existence of α-helix structures. Similarly, Ara h 2 see Fig. 8a,b also shows a characteristic positive peak at 194 nm and negative peak at 208 nm and 222 nm. The changes in the percentage of conformational units for both Ara h 1 and Ara h 2 are presented in Table 1. Thus, implying that both Ara h 1 and Ara h 2 predominantly contain α-helix and these results are consistence with previous results from Venkatratnam et al.^23^.

**Table 1:** Percentage of the secondary structure in Ara h 1 and Ara h 2 in WP and DPF.

		Control (%)	15 min (%)	30 min (%)	45 min (%)	60 min (%)
*Ara h 1*	**WP**					
	α-helix	52	50	46	39	35
	β-strands	30	27	27	29	25
	Random Coil	18	23	27	32	40
	**DPF**					
	α-helix	52	46	42	36	32
	β-strands	30	34	36	32	29
	Random Coil	18	20	22	32	39
*Ara h 2*	**WP**					
	α-helix	48	44	36	34	32
	β-strands	32	29	23	27	25
	Random Coil	20	27	39	39	43
	**DPF**					
	α-helix	48	42	39	33	26
	β-strands	32	31	33	31	36
	Random Coil	20	27	28	36	38

With increases in the plasma treatment dose, a progressive decrease in the molar ellipticity was observed for both Ara h 1 and Ara h 2 in DPF and WP. The native/control (untreated) Ara h 1 is composed of 52% α-helix, 30% β strands and 18% random coil and native/ control (untreated) Ara h 2 is 48% α-helix, 32% β strands and 20% random coil. As shown in Table 1 there was a decline in the percentage of α-helix while there is an increase in the random coils, however, β strands and turns coils exhibited a varied pattern. The α-helix in DPF for Ara h 1 decreased after 15 min treatment with a similar decrease observed for Ara h 1 in WP. On the other hand, upon plasma treatment a progressive decrease in α-helix was observed for Ara h 2 from 15 min in DPF. This change in both the positive and negative molar ellipticity show an alternation in the secondary structure of both proteins. Ekezie et al.^40^ also reported variations in the secondary structure in king prawn allergen using a cold plasma jet treatment. The oxidative interactions of plasma reactive species with protein cause alterations in the secondary structure.

Additionally, the change in the secondary structure could also be due to the aggregation thus causing modification of the antigen epitopes and their binding ability. However, the mechanism of action of plasma on the allergenicity of protein is not completely understood. Nevertheless, there are multiple known mechanisms that affect the protein structure. The active species mainly OH· or superoxide cleaves the protein into peptides and further into amino acids affecting their side chains. The dominant alteration is cleavage of disulphide bonds/oxidation of sulphur containing amino acids leading to the destruction of the binding sites for antibodies. Similar changes in the secondary structure of peanut protein isolate were reported by Ji et al.^47^. These authors suggested that the bombardment with high energetic ions resulted in a decrease in α-helix and β turns and overall destroying the orderly structure of PPI. Han et al.^43^ studied the effects of a jet plasma on activity of HRP and observed a similar change in the secondary structure. They suggested that the change in the structure could be due to the deformation of the microstructure along with the unfolding of polypeptide. The interactions of reactive species which can change the conformational structure, protein denaturation and reduction of protein solubility due to aggregate formation all of which possibly reduce the allergenicity. This study indicates the importance of the food matrix composition, type of allergen and structure of allergen protein on plasma induced effects. Furthermore, Surowsky et al.^21^ also reported significant effects on the protein structure, which was linked to the formation of hydrogen bonding leading to reduction in peptide linkages. Nonetheless, no clear consensus can be reached on the definite mechanism for the reduction of allergenicity of Ara h1/Ara h 2 by plasma. Additional work is still necessary to identify the exact interactions between plasma activate species and the allergen protein.

## Conclusion

The present study was designed to evaluate the alterations in allergenic responses and structural changes of major peanut allergens after cold plasma treatment. The results reveal a significant reduction in the antigenicity of major peanut allergens Ara h 1 and Ara h 2 for both DPF and WP. The SDS-PAGE analysis revealed reduction in protein solubility and possible formation of insoluble aggregates. Longer plasma treatments resulted in changes in the secondary structure of proteins. The modification in α-helix and β-sheets structures leads to changes in epitope binding capacity, thereby affecting its antigenicity. Cold plasma offers a promising alternative tool to decrease the allergenicity of peanut. Future work should focus on in vivo studies to verify the allergic reduction of cold plasma-treated peanut.

## Supplementary information

Supplementary file1

Supplementary file2
