# Supplementary material for: Impact of cold plasma processing on major peanut allergens
Source: Sci Rep. 2020 Oct 12;10:17038. doi: 10.1038/s41598-020-72636-w (PMC7550356; doi:10.1038/s41598-020-72636-w)
Supplement: Supplementary file 1 — Supplementary file1 [file 41598_2020_72636_MOESM1_ESM.docx]

**Impact of cold plasma processing on major peanut allergens**

**Harshitha Venkataratnam^1^, Orla Cahill^1^, Chaitanya Sarangapani^1^, PJ Cullen^1,2,3^, Catherine Barry-Ryan^1^**

**^1^**School of Food Science and Environmental Health, College of Sciences and Health,

Technological University Dublin, Cathal Brugha Street, Dublin 1, Republic of Ireland

^2^Centre for Advanced Food Enginomics, School of Chemical and Biomolecular Engineering, The University of Sydney, Sydney, Australia

^3^Plasmaleap Technologies, Merewether Building, City Road, Sydney Australia

Corresponding author **email:** [harshitha.venkataratnam@myTUDublin.ie](mailto:harshitha.venkataratnam@myTUDublin.ie)

**Supplementary figures**


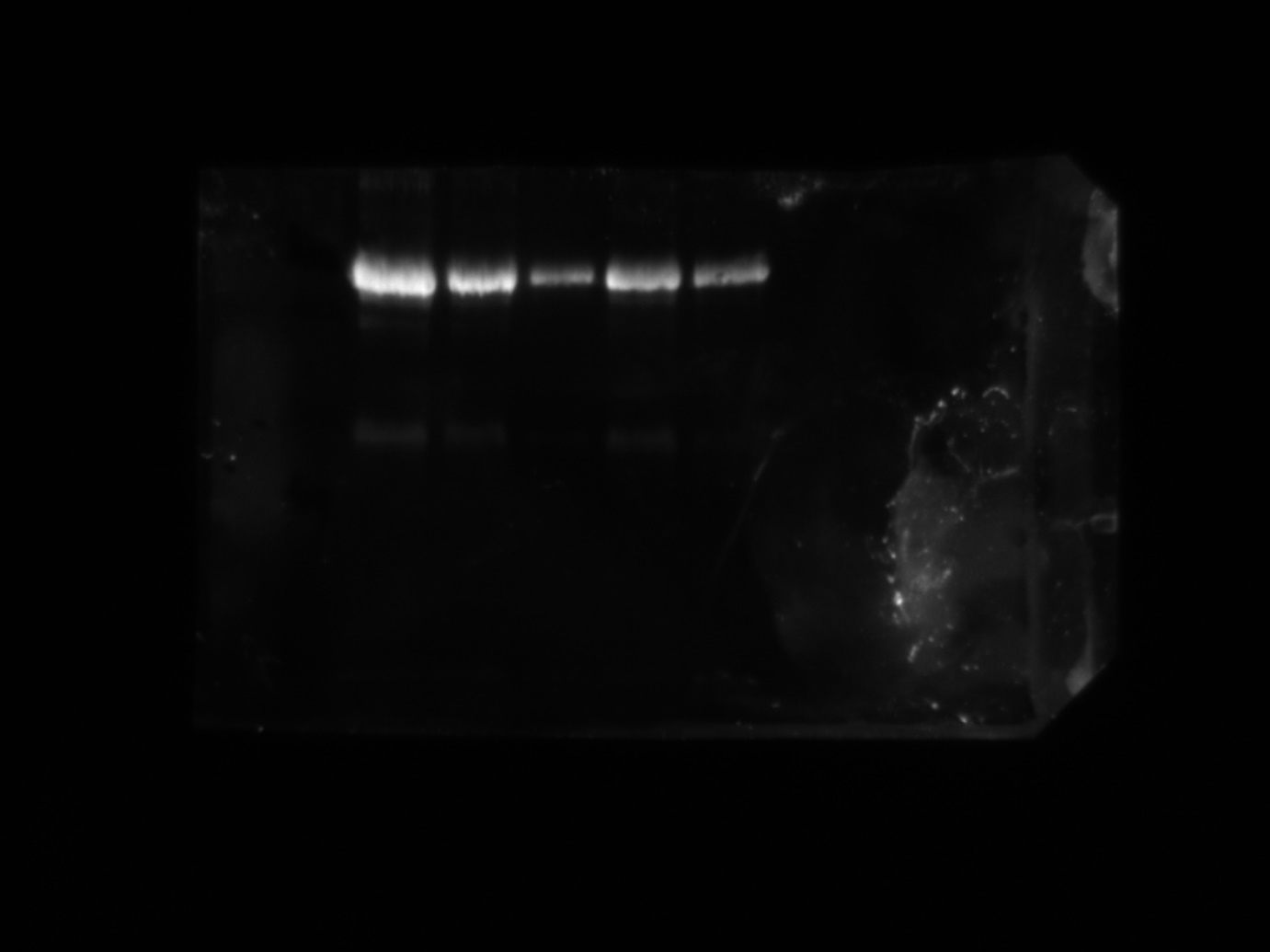


**Fig S1: Western blot analysis of Whole peanut of Ara h 1 (Note: Lane 1: Control; Lane 2- 15min; Lane 3: 60 min; Lane 4: 45 min Lane 5: 30 min)**


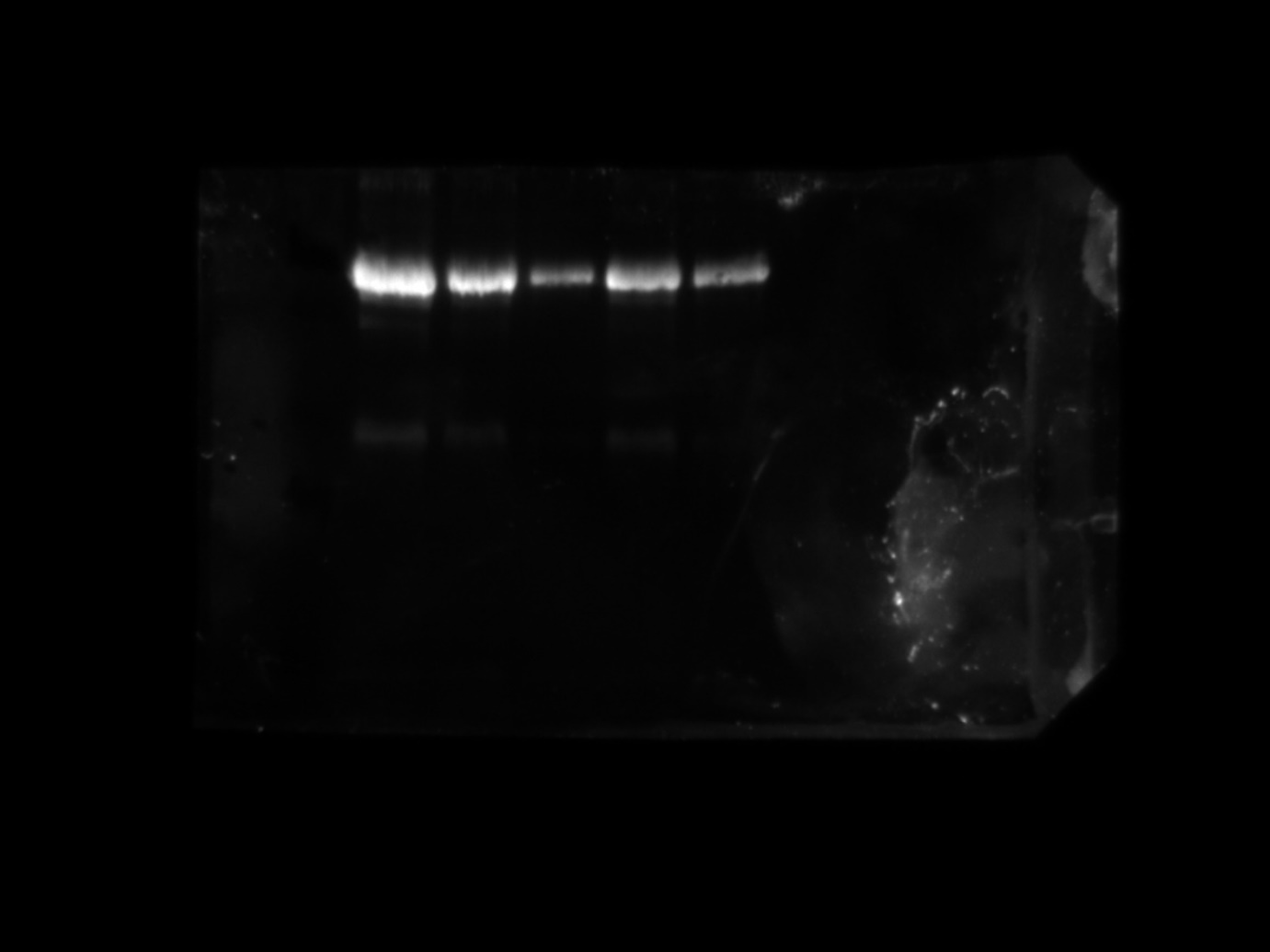


**Fig S2: Western blot analysis of Whole peanut of Ara h 1 (Note: Lane 1: Control; Lane 2- 15min; Lane 3: 60 min; Lane 4: 45 min Lane 5: 30 min)**


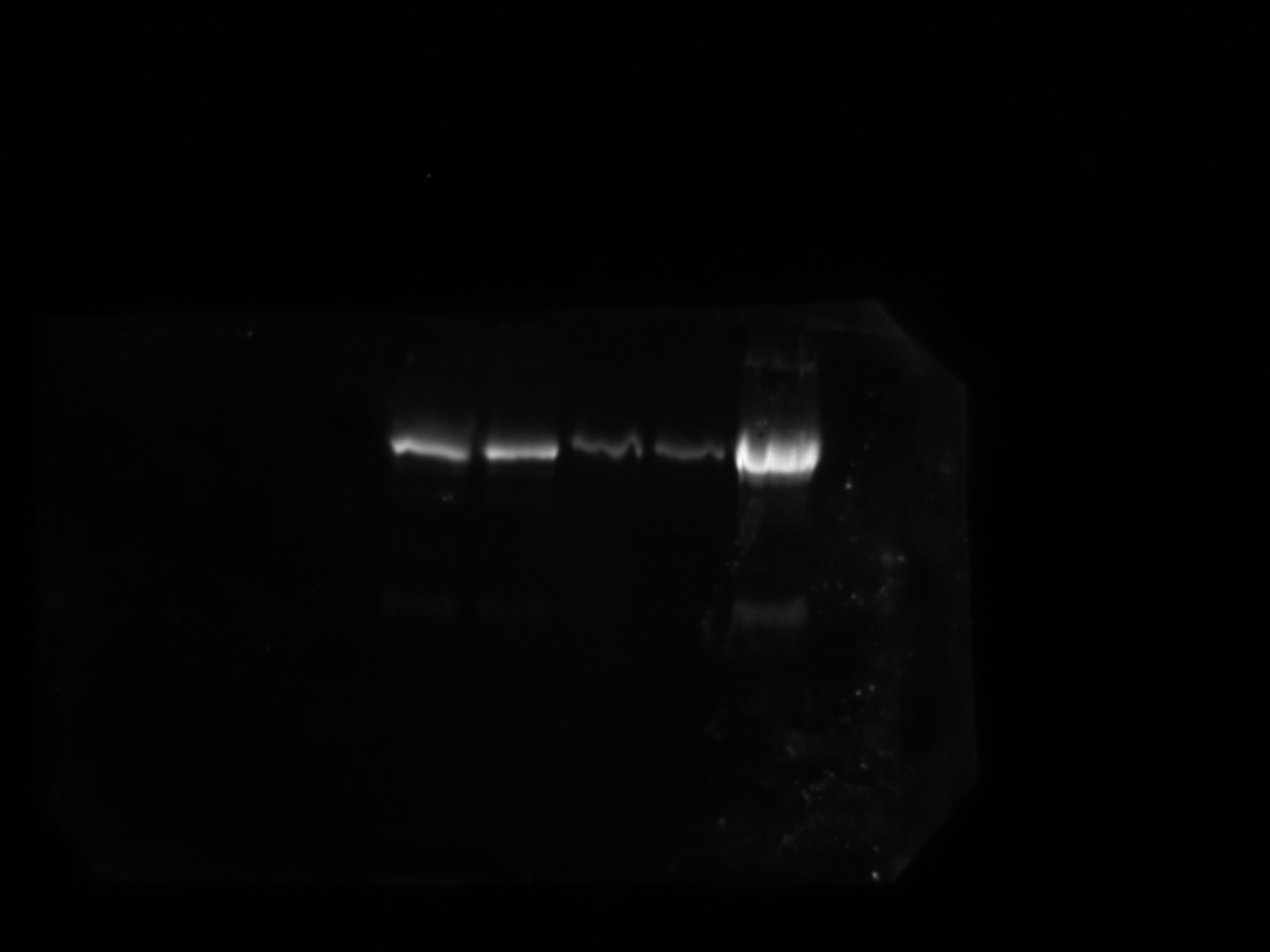


**Fig S3: Western blot analysis of Defatted peanut flour of Ara h 1 (Note: Lane 1: 15min; Lane 2:30 min; Lane 3: 45 min; Lane 4: 60 min Lane 5: control)**


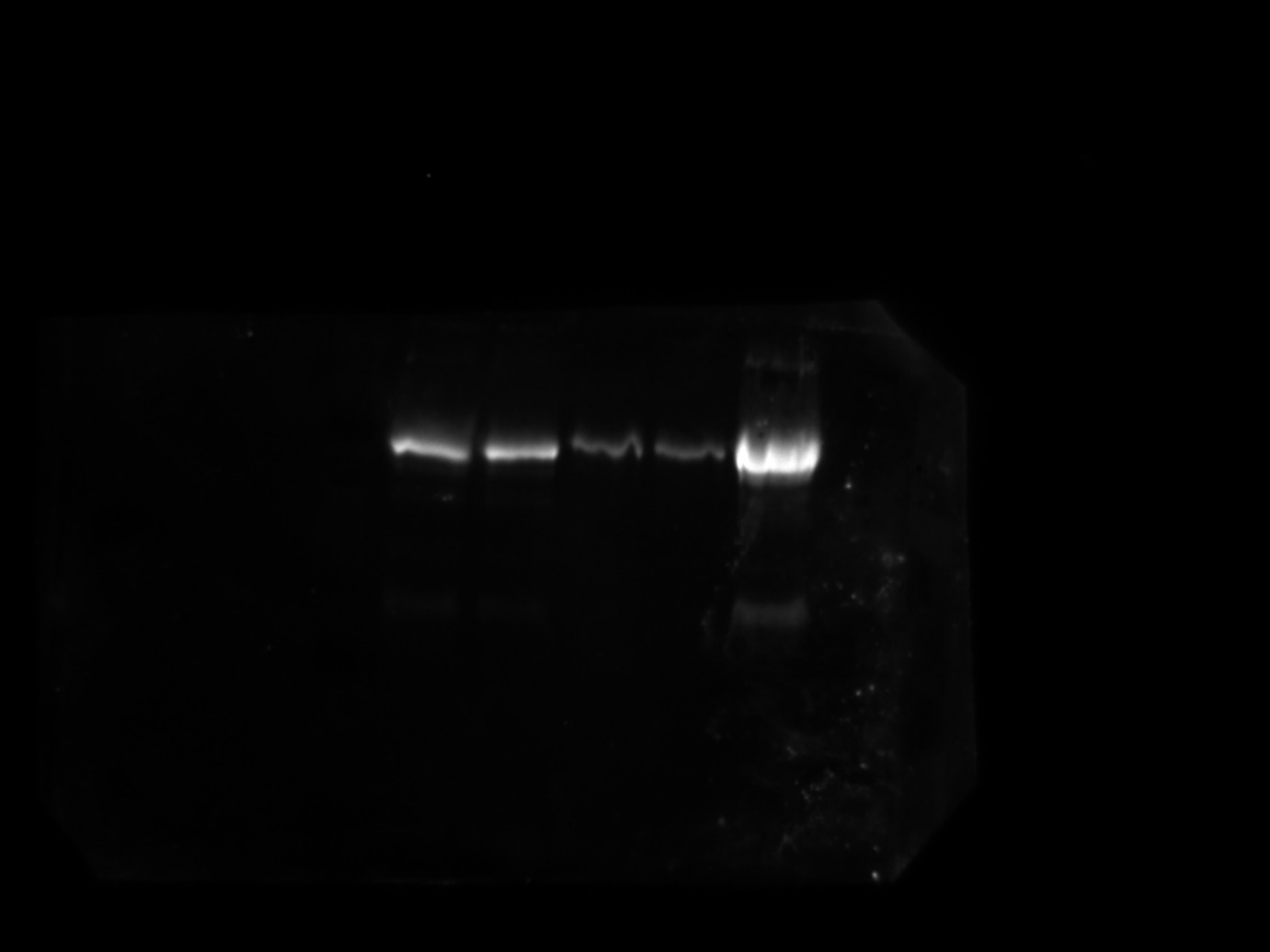


**Fig S4: Western blot analysis of Defatted peanut flour of Ara h 1 (Note: Lane 1: 15min; Lane 2:30 min; Lane 3: 45 min; Lane 4: 60 min Lane 5: control)**


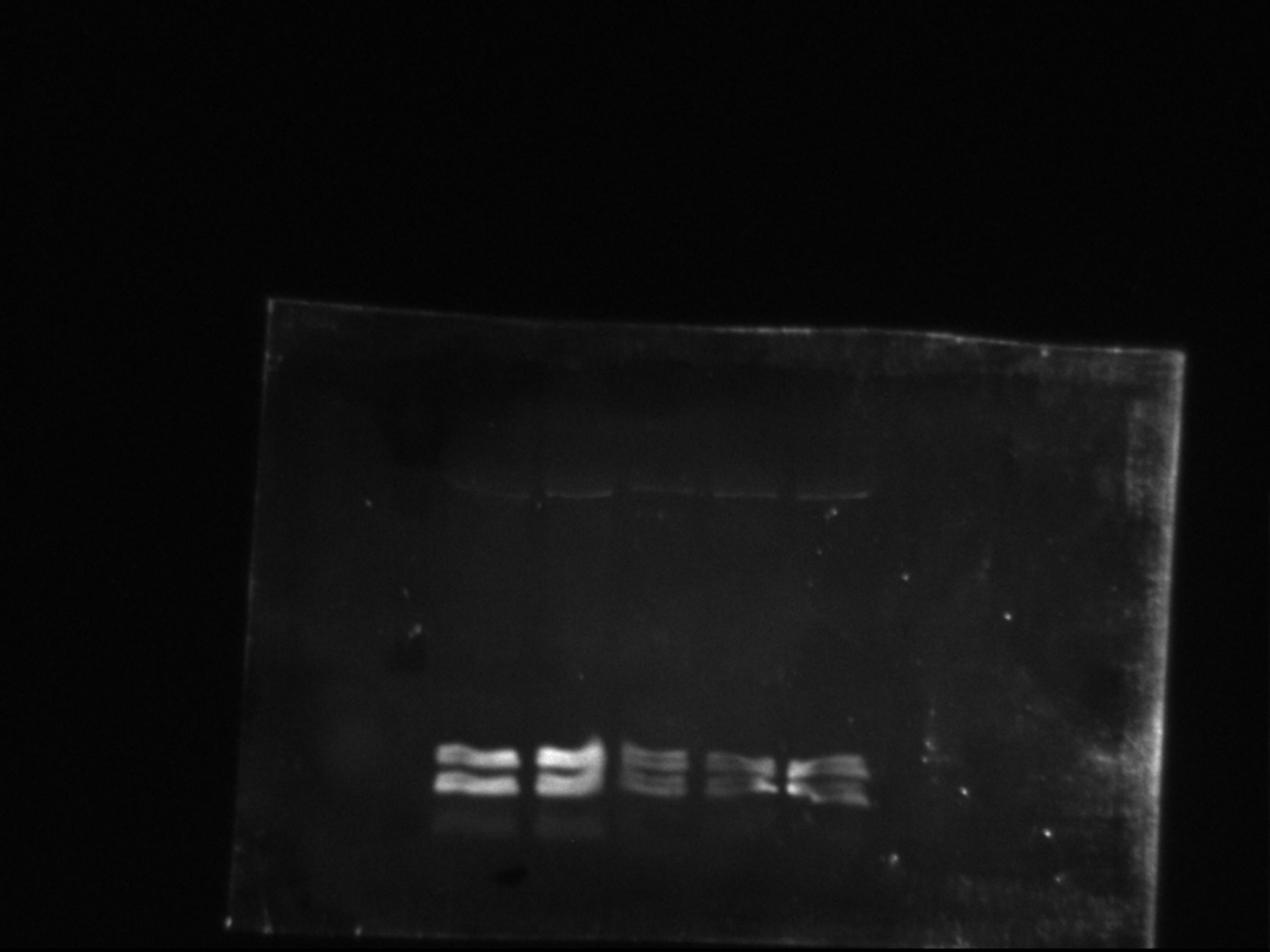


**Fig S5: Western blot analysis of Whole peanut of Ara h 2 (Note: Lane 1: Control; Lane 2- 15min; Lane 3: 30 min; Lane 4: 45 min Lane 5: 60 min)**


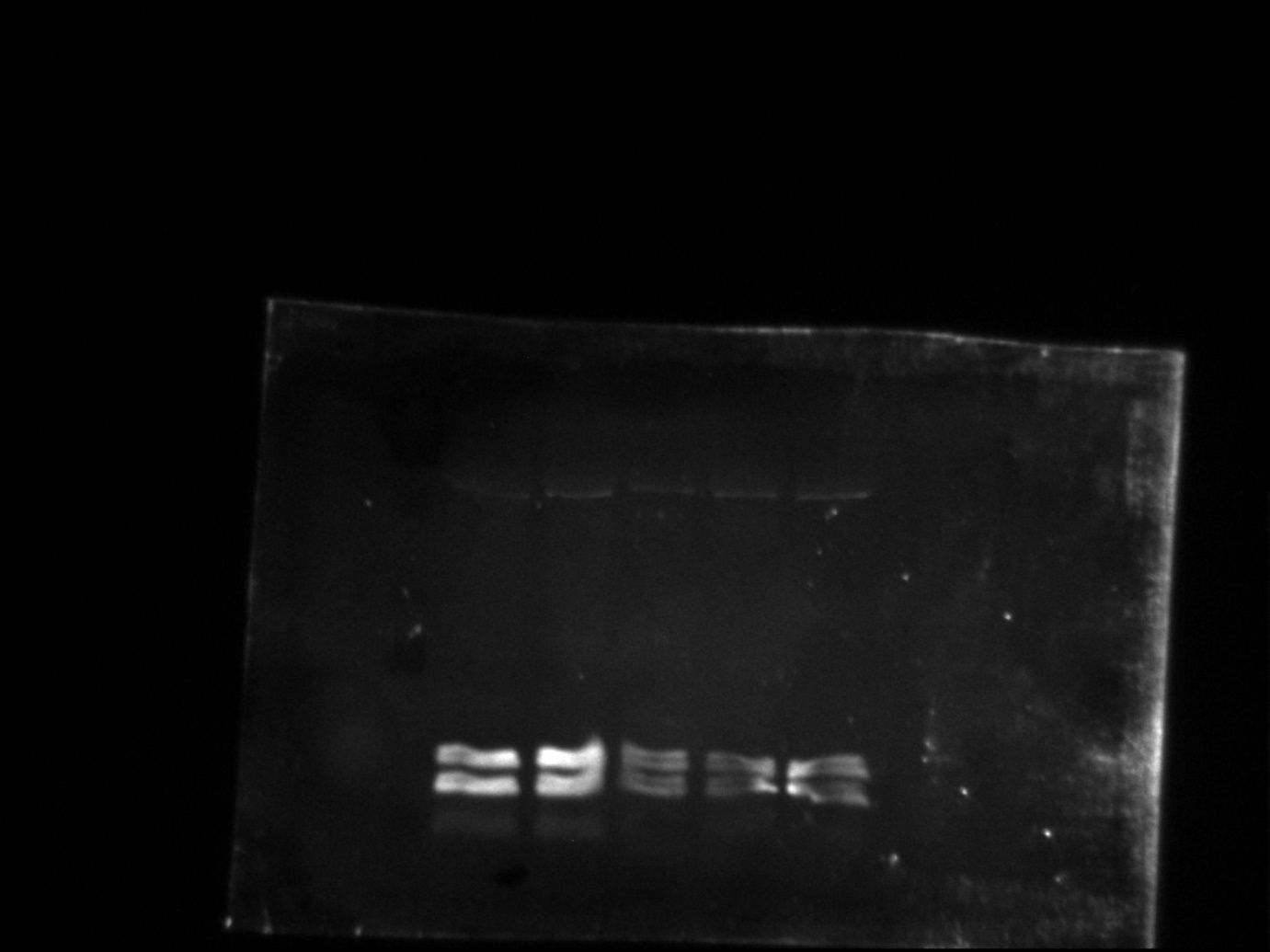


**Fig S6: Western blot analysis of whole peanut of Ara h 2 (Note: Lane 1: Control; Lane 2- 15min; Lane 3: 30 min; Lane 4: 45 min Lane 5: 60 min)**


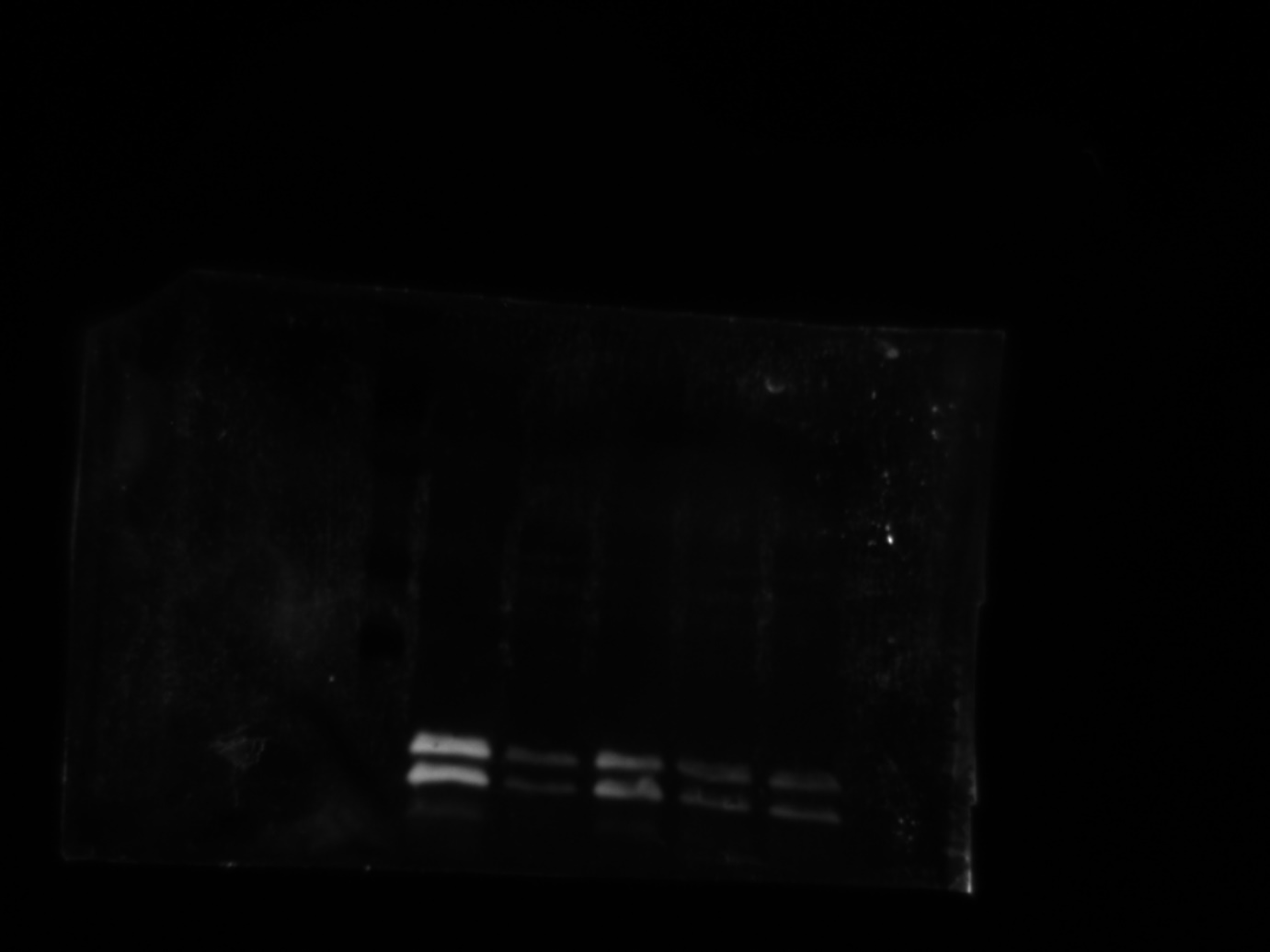


**Fig S7: Western blot analysis of Defatted peanut flour of Ara h 2 (Note: Lane 1: control; Lane 2:60 min; Lane 3: 15 min; Lane 4: 30 min Lane 5: 45 min)**


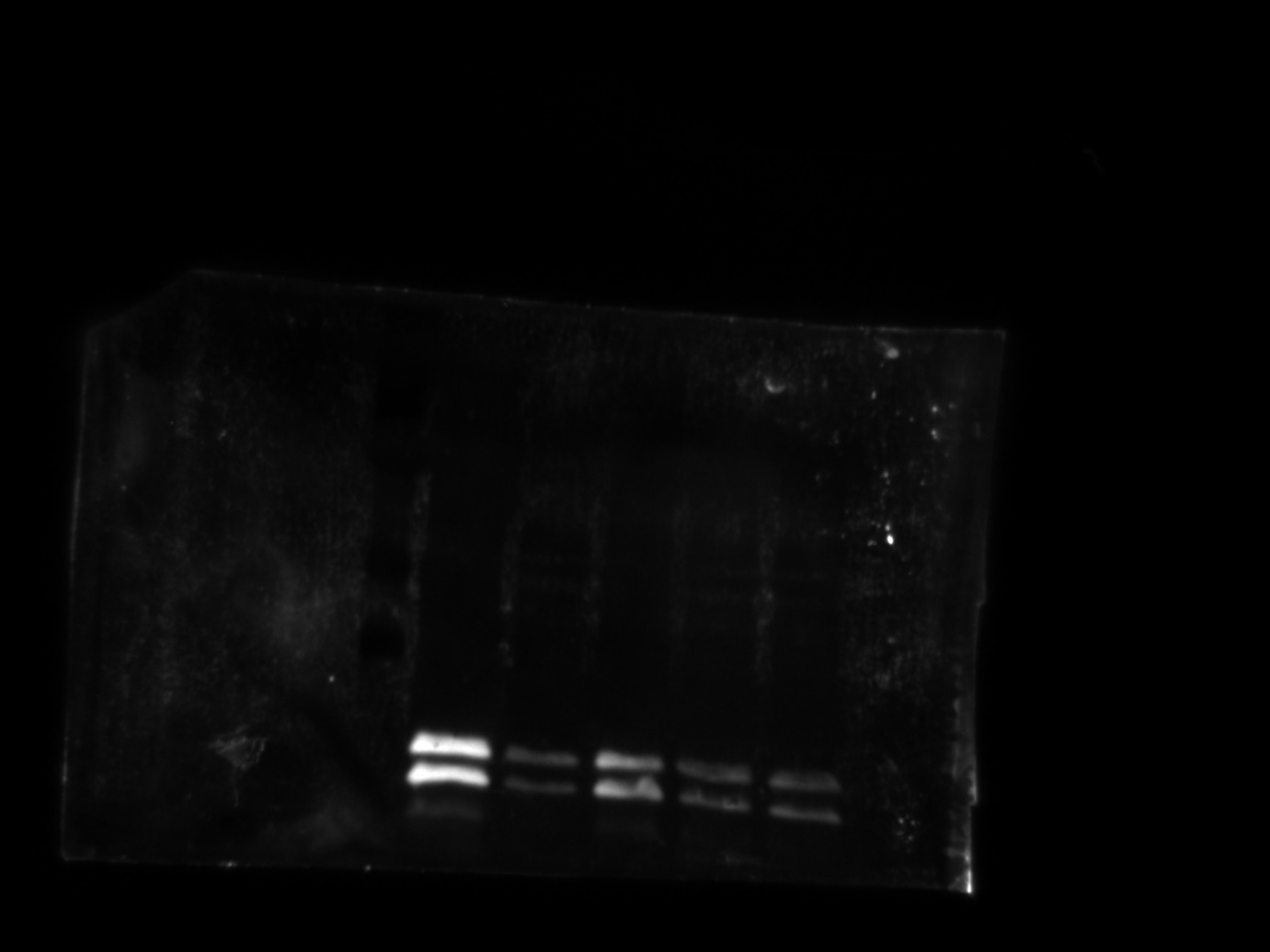


**Fig S8: Western blot analysis of Defatted peanut flour of Ara h 2 (Note: Lane 1: control; Lane 2:60 min; Lane 3: 15 min; Lane 4: 30 min Lane 5: 45 min)**
