# Supplementary material for: Impact of cold plasma processing on major peanut allergens
Source: Sci Rep. 2020 Oct 12;10:17038. doi: 10.1038/s41598-020-72636-w (PMC7550356; doi:10.1038/s41598-020-72636-w)
Supplement: Supplementary file 2 — Supplementary file2 [file 41598_2020_72636_MOESM2_ESM.docx]

**Impact of cold plasma processing on major peanut allergens**

**Harshitha Venkataratnam^1^, Orla Cahill^1^, Chaitanya Sarangapani^1^, PJ Cullen^1,2,3^, Catherine Barry-Ryan^1^**

**^1^**School of Food Science and Environmental Health, College of Sciences and Health,

Technological University Dublin, Cathal Brugha Street, Dublin 1, Republic of Ireland

^2^Centre for Advanced Food Enginomics, School of Chemical and Biomolecular Engineering, The University of Sydney, Sydney, Australia

^3^Plasmaleap Technologies, Merewether Building, City Road, Sydney Australia

Corresponding author **email:** [harshitha.venkataratnam@myTUDublin.ie](mailto:harshitha.venkataratnam@myTUDublin.ie)

**Supplementary figures**


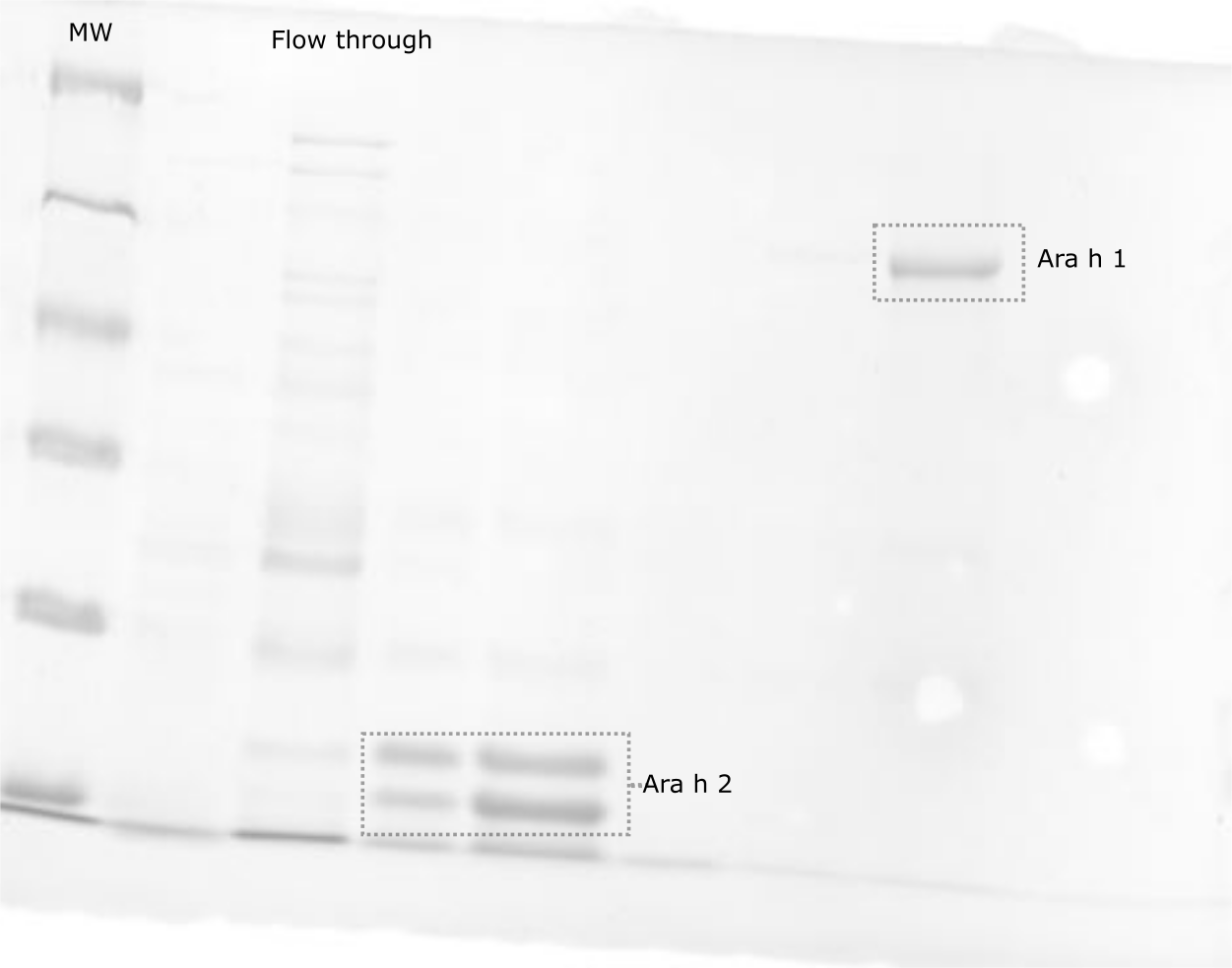


**Fig S1 R1 : Purified Ara h 1 and Ara h 2 from cold plasma treated DPF for 15 mins before desalting for CD spectroscopy measurements**


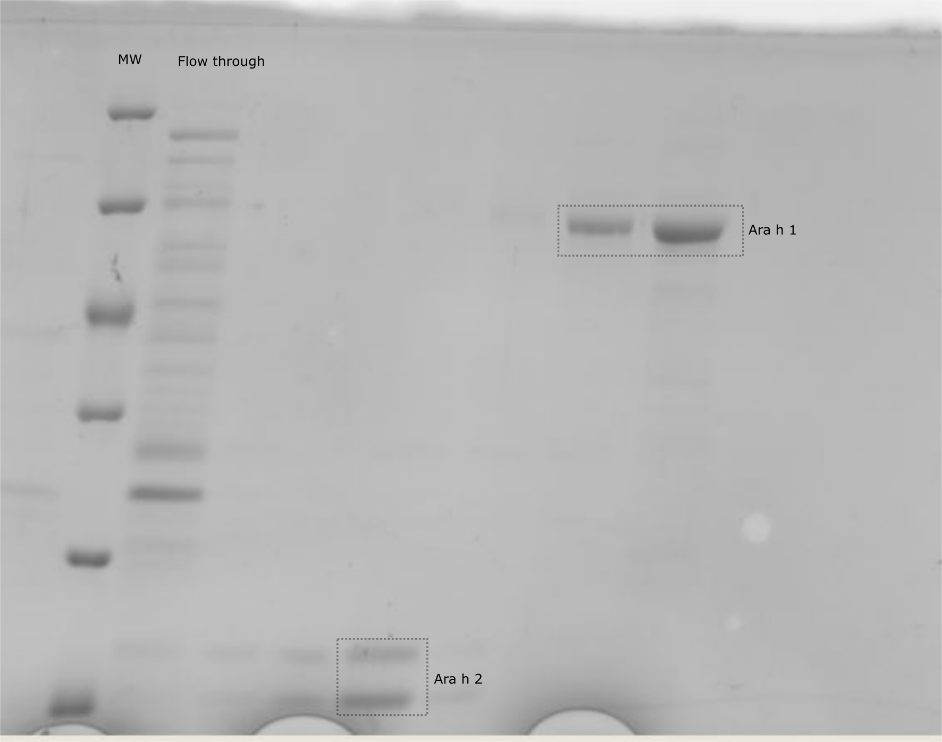


**Fig S2 R2: Purified Ara h 1 and Ara h 2 from cold plasma treated DPF for 30 mins before desalting for CD spectroscopy measurements**


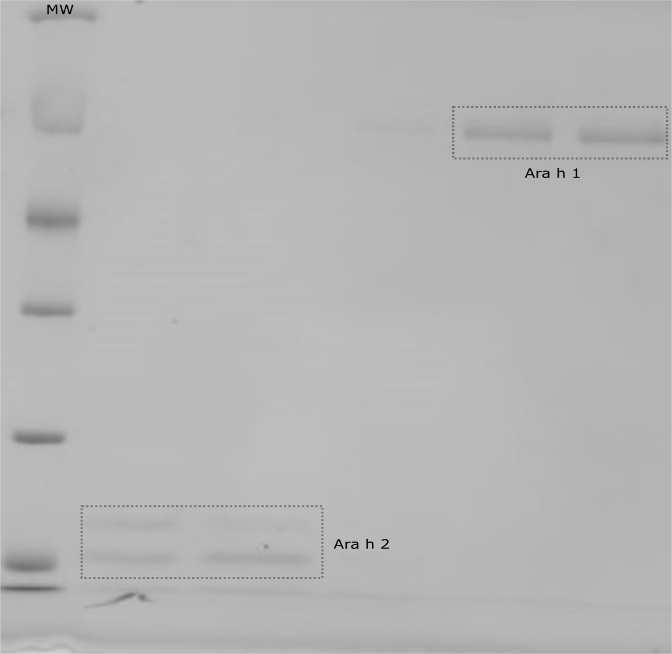


**Fig S3 R3 : Purified Ara h 1 and Ara h 2 from cold plasma treated DPF for 45 mins before desalting for CD spectroscopy measurements**


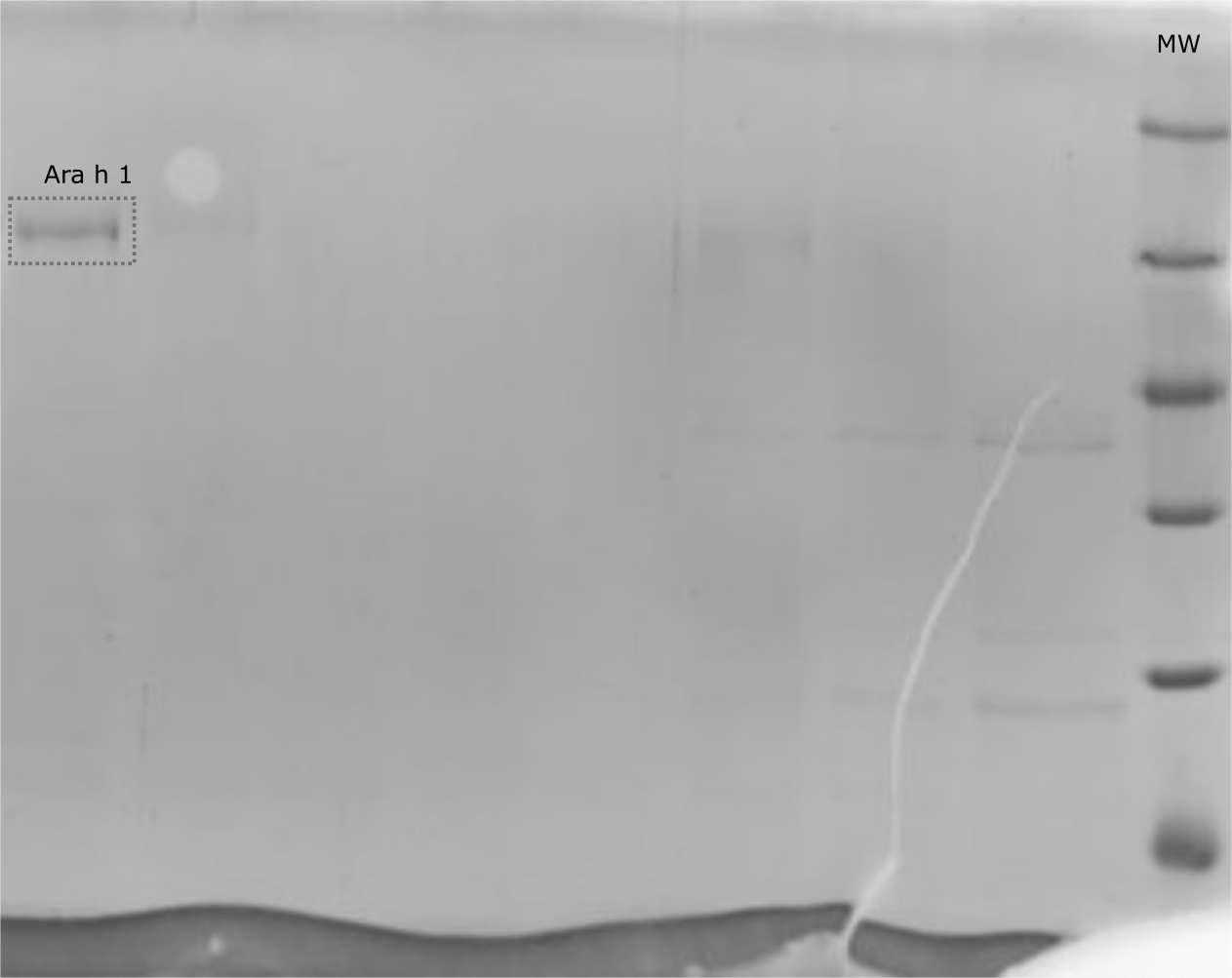


**Fig S4 R4 : Purified Ara h 1 from cold plasma treated DPF for 60 mins before desalting for CD spectroscopy measurements**


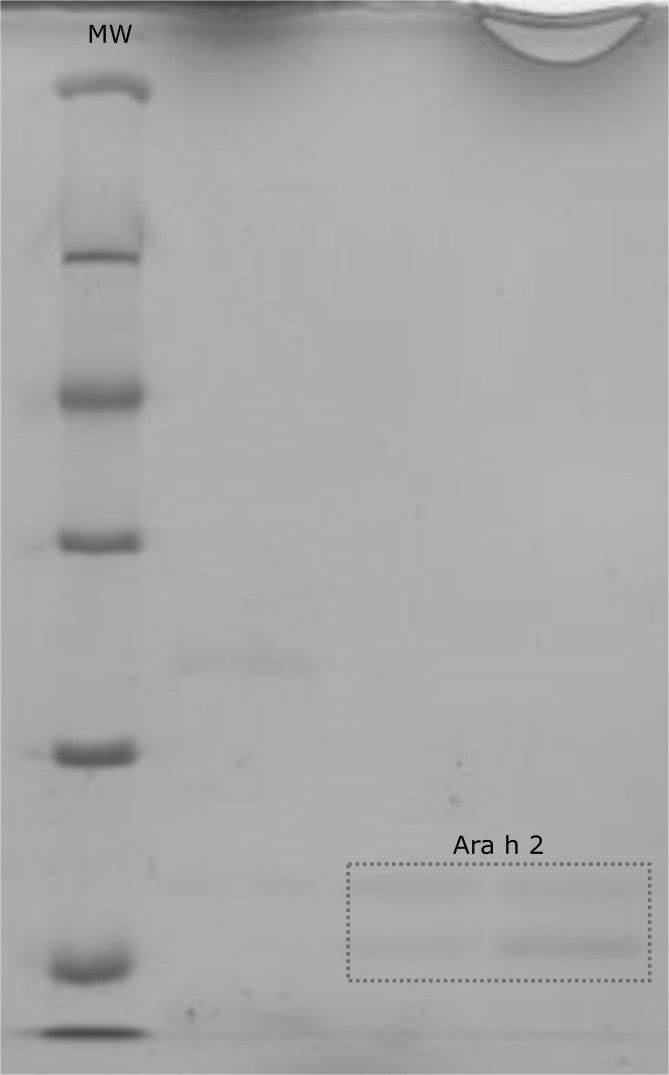


**Fig S5 R5 : Purified Ara h 2 from cold plasma treated DPF for 60 mins before desalting for CD spectroscopy measurements**
